# Relative pandemic severity in Canada and four peer nations during the SARS-CoV-2 pandemic

**DOI:** 10.14745/ccdr.v49i05a05

**Published:** 2023-05-01

**Authors:** Amy Peng, Alison Simmons, Afia Amoako, Ashleigh Tuite, David Fisman

**Affiliations:** 1 Dalla Lana School of Public Health, University of Toronto, Toronto, ON; 2 Public Health Agency of Canada, Ottawa, ON

**Keywords:** Canada, pandemic severity, SARS-CoV-2, standardization, health economics, public health

## Abstract

**Background:**

National responses to the severe acute respiratory syndrome coronavirus 2 (SARS-CoV-2) pandemic have been highly variable. We sought to explore the effectiveness of the Canadian pandemic response up to May 2022 relative to responses in four peer countries with similar political, economic and health systems, and with close historical and cultural ties to Canada.

**Methods:**

We used reported age-specific mortality data to generate estimates of pandemic mortality standardized to the Canadian population. Age-specific case fatality, hospitalization, and intensive care admission probabilities for the Canadian province of Ontario were applied to estimated deaths, to calculate hospitalizations and intensive care admissions averted by the Canadian response. Health impacts were valued in both monetary terms, and in terms of lost quality-adjusted life years.

**Results:**

We estimated that the Canadian pandemic response averted 94,492, 64,306 and 13,641 deaths relative to the responses of the United States, United Kingdom and France, respectively, and more than 480,000 hospitalizations relative to the United States. The United States pandemic response, if applied to Canada, would have resulted in more than $40 billion in economic losses due to healthcare expenditures and lost quality-adjusted life years. In contrast, an Australian pandemic response applied to Canada would have averted over 28,000 additional deaths and averted nearly $9 billion in costs.

**Conclusion:**

Canada outperformed several peer countries that aimed for mitigation rather than elimination of SARS-CoV-2 in the first two years of the pandemic, with substantial numbers of lives saved and economic costs averted. However, a comparison with Australia demonstrated that an elimination focus would have saved Canada tens of thousands of lives as well as substantial economic costs.

## Introduction

The global severe acute respiratory syndrome coronavirus 2 (SARS-CoV-2) pandemic has taken a fearsome toll on mortality, life expectancy and population health globally, but not all countries have been impacted equally. The reasons for this heterogeneity are only partly understood. Population age structure is a key contributor to SARS-CoV-2 severity ((1,2)); however, countries with older age distributions (such as Japan) have been less severely affected than its high-income peers ((3)). Japan’s early focus on the airborne nature of SARS-CoV-2, and the widespread acceptance of masking, may also have been important mitigators ((3,4)). Marked heterogeneity in severity was seen across countries that have similar age structures but were slow to recognize airborne transmission of SARS-CoV-2.

A case in point is the differential severity of the pandemic in Canada and the United States (US); both are wealthy, federal democracies with advanced medical care systems. In both countries, the coronavirus disease 2019 (COVID-19) pandemic has had a major impact on population health and the economy. The similarities and differences between the two countries’ healthcare systems have made cross-national comparisons an important source of insight into the strengths and weaknesses of their respective health systems ((5)). During the COVID-19 pandemic, both COVID-19 cases and deaths per capita have been substantially higher in the US than in Canada ((6)). Australia represents another reasonable peer for Canada for comparison purposes. Australia is similar to Canada in terms of income, culture and governance, but employed more stringent pandemic control measure and consequently had much lower per capita SARS-CoV-2 pandemic mortality as of May 2022 ((7)). The United Kingdom (UK) and France share ties of economy, culture and history with Canada (as hubs of the British Commonwealth and La Francophonie, both of which include Canada), and may also represent appropriate comparators.

Debate in the Canadian public sphere around pandemic policy has often focussed on whether Canada’s approach to disease control should have been more or less stringent. Assuming that differences in outcomes were at least partly driven by policy rather than the independent actions and choices of individuals, we sought to explore the differences in outcomes that Canada would have experienced over the first two years of the SARS-CoV-2 pandemic had it followed the path of the US, the UK, France or Australia. We had previously performed such an analysis in March 2021, with comparison restricted to Canada and the US ((6)). While our objective was not to perform a formal cost-utility analysis of the Canadian pandemic response relative to responses in these peer nations, the question of costs averted, or excess costs accrued, both through hospitalizations and premature loss of life, is an important one, and we incorporated simple valuations of these quantities into our analysis. These may help inform future cost-utility analyses on this question.

## Methods

We obtained national COVID-19-attributed death estimates from Public Health Agency of Canada, and national health authorities for the US, the UK, France and Australia until late April or early May of 2022, as available ((7–11)). We chose these countries as comparator peers because all are high income countries with advanced health systems, and all have strong cultural, political, and historical links to, and similarities with Canada. Of these five countries, all but Australia ((12,13)) sought to mitigate rather than eliminate SARS-CoV-2 during the first two years of the pandemic. Some Canadian provinces and territories, notably Atlantic provinces and Northern Territories ((14)) did pursue elimination at times. Population estimates were obtained from national census agencies for all countries ((15–19)). We calculated the number of excess or deficit deaths that would have been expected in Canada under approaches employed in peer countries using direct standardization ((20)). Because country death data were reported using slightly different age groupings, we reallocated Canadian deaths to mirror the distribution of SARS-CoV-2 deaths, by two-year age increments, due to data availability in the province of Ontario (available to January 18, 2022). Deaths were assumed to be equally distributed between years in each two-year category. Standardized mortality ratios (SMR) for Canada, relative to other countries, were estimated by dividing observed by expected deaths (i.e. the deaths that would have occurred with a US, UK, France or Australia-equivalent response). The 95% confidence limits for SMR were calculated by estimating standard errors as (1/A+1/B)^1/2^, where A and B are death counts in each of the two peer countries, as described previously ((20)).

Observed deaths were subtracted from expected deaths to calculate deaths averted. We divided averted deaths by age-specific case-fatality estimates from Ontario to estimate averted cases. We applied age-specific risks of hospital admission and intensive care admission, derived from Ontario case data, to calculate hospital and intensive care admissions averted. We placed a monetary value on hospitalizations and intensive care unit (ICU) admissions averted based on Canadian cost estimates generated by the Canadian Institute for Health Information ((21)). The approach of Briggs *et al.*, modified for the Canadian context by Kirwin *et al.*, was used to estimate quality-adjusted life years (QALY) lost for deaths occurring in each age group ((22,23)). We monetized QALY losses averted by applying a net expected benefit approach, with QALY valued at $30,000 as per Kirwin *et al.* ((23)). We compared the stringency of pandemic responses using the Oxford Government Coronavirus Response Tracker’s Pandemic Stringency Index ((24)). The stringency was plotted against time and differences in the stringency between Canada and other countries were evaluated with the Wilcoxon rank-sum test. All input data are publicly available.

## Results

Fewer SARS-CoV-2-related deaths per capita had occurred in Canada than in the US in all age groups as of May 2022, with SMR significantly less than one for all age groups in Canada. A similar pattern was seen when Canada was compared to the UK, except in children aged 0–14 years, where there was no significant difference between the two countries (SMR 1.02, 95% CI: 0.67–1.55). In comparison with France, Canada experienced significantly fewer deaths per capita in adults aged 40–89 years, more deaths than France in those aged 20–29 years and 90 years and older, and no difference in those younger than 20 years. In comparison with Australia, Canada had significantly higher SARS-CoV-2-related deaths per capita in all age groups except those aged 10–19 years, where differences were not significant (SMR 2.24, 95% CI: 0.81–6.16) (**Table 1**).

**Table 1:** Standardized mortality ratios for the first two years of the SARS-CoV-2 pandemic in peer countries compared to Canada

Age group (years)	Deaths	Population	Cumulative mortality per 1,000	Expected deaths, Canadian population	Observed Canadian deaths^a^	Standardized mortality ratio	95% CI
**United States**							
0–17	1,045	73,284,400	0.01	103.42	37	0.35	0.25–0.49
18–29	6,257	52,870,600	0.12	700.11	136	0.19	0.16–0.23
30–39	18,148	43,375,000	0.42	2,244.47	315	0.14	0.13–0.16
40–49	42,961	39,929,000	1.08	5,265.77	660	0.13	0.12–0.14
50–64	187,272	62,110,000	3.02	23,329.55	3,772	0.16	0.16–0.17
65–74	229,682	3,1487,000	7.29	29,816.49	6,422	0.22	0.21–0.22
75–84	257,553	15,407,000	16.72	35,486.56	10,899	0.31	0.30–0.31
85 and over	255,780	5,893,000	43.40	37,823.67	18,038	0.48	0.47–0.48
Total	991,396	324,356,000	-	134,770	40,278	-	-
**United Kingdom**							
0–14	64	11,974,857	0.005	32	33	1.02	0.67–1.55
15–44	2,748	25,311,086	0.109	1,631	685	0.42	0.39–0.46
45–64	21,139	17,286,653	1.223	12,378	4,466	0.36	0.35–0.37
65–74	30,745	6,719,287	4.576	18,703	6,491	0.35	0.34–0.36
75–84	59,945	4,129,982	14.515	30,812	21,317	0.69	0.68–0.70
85 and over	78,125	1,659,369	47.081	41,028	7,286	0.18	0.17–0.18
Total	192,766	67,081,234	-	104,584	40,278	-	-
**France**							
0–9	37	7,706,041	0.005	19	29	1.54	0.95–2.50
10–19	31	8,421,914	0.004	15	15	0.98	0.53–1.82
20–29	147	7,525,983	0.020	99	128	1.29	1.02–1.63
30–39	465	8,279,577	0.056	301	315	1.05	0.91–1.21
40–49	1,337	8,572,713	0.156	763	660	0.87	0.79–0.95
50–59	4,576	8,813,899	0.519	2,664	1,862	0.70	0.66–0.74
60–69	13,344	8,000,803	1.668	8,074	4,349	0.54	0.52–0.56
70–79	26,358	5,959,261	4.423	13,862	8,633	0.62	0.61–0.64
80–89	43,387	3,214,055	13.499	18,460	13,844	0.75	0.74–0.76
90 and over	25,895	927,995	27.904	9,662	10,443	1.08	1.06–1.11
Total	115,577	67,422,241	-	53,919	40,278	-	-
**Australia**							
0–9	8	3,156,780	0.003	10	29	2.91	1.33–6.37
10–19	5	3,097,360	0.002	7	15	2.24	0.81–6.16
20–29	22	3,476,779	0.006	32	128	3.97	2.53–6.24
30–39	65	3,780,122	0.017	92	315	3.41	2.61–4.46
40–49	124	3,294,734	0.038	184	660	3.58	2.96–4.34
50–59	322	3,143,647	0.102	526	1,862	3.54	3.15–3.99
60–69	726	2,737,883	0.265	1,284	4,349	3.39	3.13–3.66
70–79	1,579	1,952,572	0.809	2,534	8,633	3.41	3.23–3.59
80–89	2,695	876,320	3.075	4,205	13,844	3.29	3.16–3.43
90 and over	1,925	221,945	8.673	3,003	10,443	3.48	3.31–3.65
Total	7,471	25,738,142	-	11,878	40,278	-	-

Abbreviations: CI, confidence intervals; SARS-CoV-2, severe acute respiratory syndrome coronavirus 2; -, not applicable

^a^ Due to redistribution of deaths to comparator peer country age categories, fractional deaths were calculated; all deaths rounded to the nearest whole number

When compared to the US, UK and France’s SARS-CoV-2 responses, we estimated that Canada’s response prevented 94,492 (95% CI: 93,593–95,360), 64,306 (95% CI: 63,394–65,189) and 13,641 (95% CI: 12,489–14,735) deaths, respectively. In contrast, an Australian response applied to Canada would have saved 28,400 (95% CI: 26,097–30,939) lives of the total number of Canadians (n=40,278) that had been lost to SARS-CoV-2 as of May 2022 (**Table 2**).

**Table 2:** Health outcomes and costs^a^ averted in peer countries compared to Canada

Outcome	Comparator peer country							
	United States	95% CI	United Kingdom	95% CI	France	95% CI	Australia^b^	95% CI
Deaths averted	94,492	93,593–95,360	64,306	63,394–65,189	13,641	12,489–14,735	−28,400	−30,939–−26,097
Hospitalizations averted	483,009	465,046–516,497	196,611	184,256–209,756	39,367	26,213–50,528	−83,281	−110,498–−67,197
ICU admissions averted	108,157	99,635–117,714	40,131	37,002–43,514	8,984	6,873–10,683	−15,335	−20,059–−12,380
QALY gained	1,060,180	943,164–1,172,874	569,981	514,483–635,306	133,517	107,018–158,498	−231,100	−277,758–−191,373
Hospitalization costs averted	10.73	10.32–11.47	4.37	4.09–4.66	0.87	0.59–1.13	−1.85	−2.42–−1.49
ICU costs averted	5.18	4.78–5.65	1.92	1.77–2.08	0.43	0.33–0.51	−0.73	−0.95–−0.59
Hospitalization costs averted (non-ICU)	5.55	5.55–5.81	2.45	2.31–2.58	0.44	0.25–0.62	−1.12	−1.46–−0.90
Net benefit of QALY gained	31.81	28.29–35.19	17.10	15.43–19.06	4.01	3.26–4.74	−6.93	−8.00–−5.50
Total costs averted	42.54	38.62–46.65	21.47	19.52–23.71	4.88	3.83–5.88	−8.78	−10.77–−7.21

Abbreviations: CI, credible intervals derived via simulation; ICU, intensive care unit; QALY, quality-adjusted life year

^a^ All costs are in billions of $CND

^b^ Negative values denote excess health consequences and costs in Canada relative to Australia

**Figure 1:**
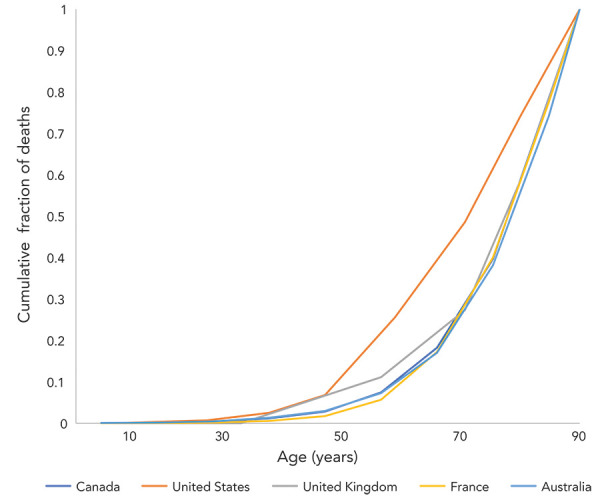
Cumulative proportion of COVID-19-attributable death by age^a^, March 2020 to May 2022 Abbreviation: COVID-19, coronavirus disease 2019 ^a^ Ages represent the midpoints of age categories. For the oldest age categories in Canada (80 years of age and over) and the United States (85 years of age and over) we assigned an age of 90 years

**Figure 2:**
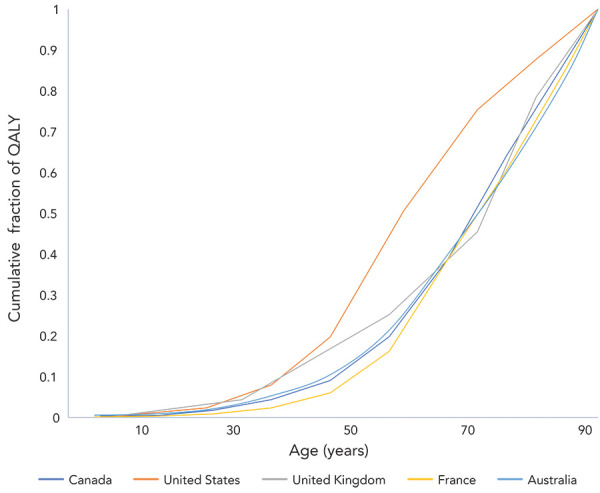
Cumulative proportion of COVID-19-attributable quality-adjusted life years loss, by age^a^, March 2020 to May 2022 Abbreviations: COVID-19, coronavirus disease 2019; QALY, quality-adjusted life years ^a^ Ages represent the midpoints of age categories. For the oldest age categories in Canada (80 years of age and over) and the United States and United Kingdom (85 years of age and over) we assigned an age of 90 years; for Australia and France, the highest age category (90 years of age and over) was assigned a value of 90 years

Distributions of deaths by age differed markedly between the US and the other countries analyzed. For example, half of deaths in the US occurred in individuals under the age of 55 years; in other countries, half of the fatalities occurred in those under approximately 75 years of age with the remainder occurring in those 75 years of age and over (**Figure 1**). A similar divergence between the US response and those in other countries was seen when we applied age-specific QALY losses to death data (**Figure 2**).

We estimated that Canada’s response saved over one million QALYs, nearly 500,000 hospitalizations and over 100,000 ICU admissions relative to what would have occurred with a response equivalent to that seen in the US (Table 2). The value of QALY losses and hospitalizations averted is estimated to be approximately $43 billion, with $32 billion due to aversion of lost QALY and the remainder due to averted hospitalizations. The Canadian response also saved QALY and averted hospitalizations and ICU admissions relative to UK and French responses. When compared to the Australian response, Canada’s response was estimated to have resulted in approximately 230,000 additional QALY lost, over 80,000 excess hospital admissions and over 15,000 excess ICU admissions as of May 2022, representing a loss of $8.78 ($7.21 to $10.77) billion (Table 2). Age-specific estimates of deaths, healthcare utilization and costs averted for each of the four peer comparator countries are presented in Table 2.

**Figure A1:**
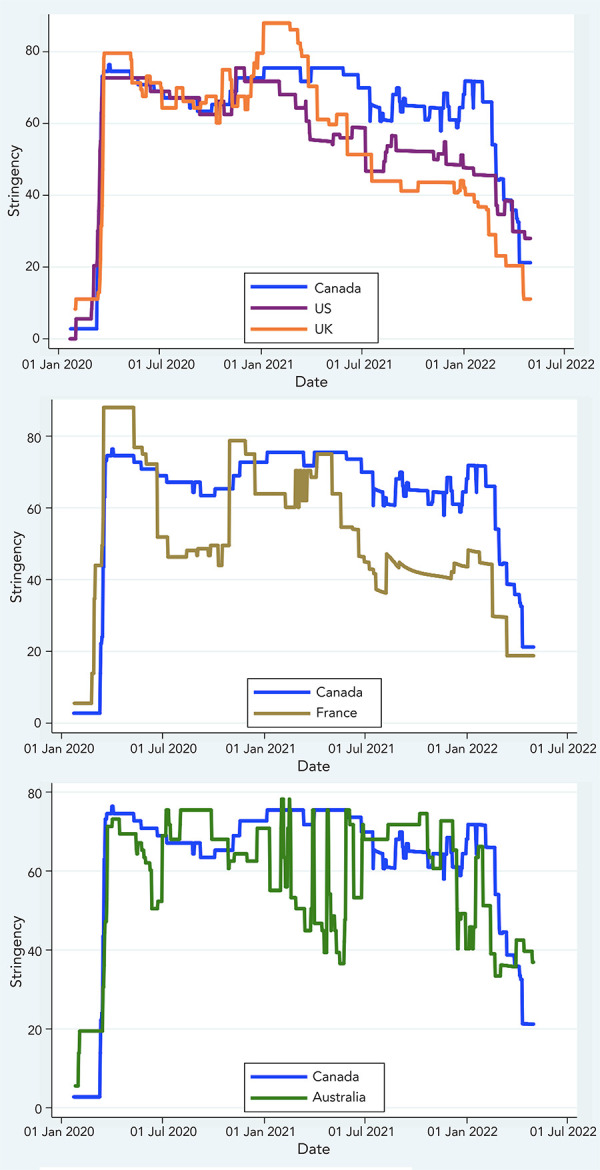
Oxford Pandemic Stringency Index by date, Canada and comparator peer countries^a^ Abbreviations: UK, United Kingdom; US, United States ^a^ Stringency values plotted to May 1, 2022. Higher values indicate more stringent control measures

The stringency of the Canadian pandemic response from March 1, 2020, to May 1, 2022, was significantly higher than the stringency in the US, the UK and France, and was also higher than the Australian stringency (*p*<0.001 for all comparisons) (**Appendix**, **Table A1** and **Figure A1**).

## Discussion

The cultural similarities and integrated economies of Canada and the US, which also have very different health systems, has long encouraged comparative research between these two countries ((5,25–27)). During the current SARS-CoV-2 pandemic, this type of research has continued, spurred, in part, by the remarkable difference in the pandemic’s impact on the two countries ((28)). Here, we demonstrate that application of age-specific US data to Canada resulted in a far deadlier pandemic in the US, with a more than three-fold higher total deaths relative to those that had occurred in Canada as of May 2022. A challenge with this type of comparison is that the US’s pandemic response has emerged as a global outlier, with SARS-CoV-2 taking a far greater toll in terms of loss of life than in any other high-income peer country. The outlier status of the US ((28)) has the effect of making Canada-US comparisons predictable in result, perhaps unfairly elevating the effectiveness of the Canadian pandemic response. As such, we also evaluated Canada’s response relative to the UK, France and Australia, which given cultural, political, economic and historical similarities to Canada, are also fair comparators.

We find that, as with the US, application of the UK’s pandemic response to Canada would have resulted in tens of thousands of additional deaths, as well as billions of dollars in excess economic losses. While Canada appears to have outperformed France as well, differences in pandemic repercussions between these two countries were more modest. In contrast, Australia emerges as a model of what Canada might have achieved by taking a more aggressive stance on disease control during the first two years of the SARS-CoV-2 pandemic. Indeed, we estimate that over 75% of Canadian pandemic deaths to date could have been averted through an Australian response, with cost savings of approximately $10 billion.

Our work complements that of Razak *et al.*, who also found that Canada had outperformed most of its G10 peers (except for Japan) with respect to pandemic-attributable mortality ((29)). However, the use of standardization, as applied here, allows us to see that the Canadian approach was far more effective than the US and UK approaches in preventing deaths in younger adults, with consequently greater gains in quality-adjusted survival. As public health and government officials in these five countries likely had access to similar information for decision-making, differences in outcomes likely reflected active policy choices. The complexity of the pandemic, and societal responses to it, make identification of causal factors challenging. Galvani *et al.* noted that a key difference between Canada and the US may relate to universal public healthcare in the former ((28)); however, universal public healthcare is also available in the UK, France and Australia. Razak *et al.* noted that Canada outperformed many high-income peer countries on vaccination ((29)). We have also suggested that cultural differences between countries, including differences in social capital and trust in government, may be important ((30)).

While Canada’s pandemic response, as reflected in the Oxford Stringency Index, was more stringent on average than the responses in the US, the UK and France, it was also more stringent than Australia’s, suggesting that stringency alone cannot explain differences in outcomes. Data from Aknin *et al.* suggest that it may not have been stringency, but the decision to aim for elimination rather than mitigation, which resulted in the low stringency and low deaths seen in countries like Australia ((31)). Although more aggressive pandemic control strategies have been criticized over perceived negative mental health impacts, Aknin *et al.* also demonstrated that the impact of excess pandemic deaths far outweighed the impact of public health interventions as a driver of negative mental health effects during the pandemic ((31)). This suggests that Canada’s approach, in addition to saving more lives and reducing more costs than US and UK responses, may have been more protective of population mental health. More stringent control strategies have also been criticized as resulting in greater negative economic impacts, and indeed Canada’s GDP declined by 1.6% in the first two years of the pandemic ((29)); however, the $43 billion Canada effectively gained by avoiding a US-style pandemic response represents over 2% of Canadian GDP (valued at around $2.1 trillion $CDN).

## Limitations

Our analysis has three key limitations. We have not attempted to capture consequences or costs of the pandemic on mental health. It should be noted that Aknin *et al.* ((31)) found that a pandemic elimination rather than mitigation stance decreased overall stringency and mental health impacts. Other important costs and impacts that we did not include, and which would likely further widen the gap in health and economic consequences between these peer countries, include disutility and lost earnings associated with hospitalization, long-term costs of chronic disease, including cardiac, respiratory and neurological disease, in those who survive SARS-CoV-2 infection, and the health, economic and societal impacts of parental loss due to the pandemic ((32–35)). As we have included only QALY gains and losses associated with death, and not incorporated those associated with short-term illness and hospitalization, or with the post-acute COVID syndrome (commonly referred to as “long COVID”), our estimates for QALY lost represent lower bounds for all countries ((36)). A second limitation of our analysis is our use of Ontario-specific case fatalities and hospitalization and intensive care admission risks to estimate outcomes averted at a national level. We use these data for pragmatic reasons: they were the most complete and granular Canadian death data to which we had access. Furthermore, Ontario’s epidemiology is likely similar to that of Canada overall, both because of similarities in demographics and health systems across the country, and also because the population of Ontario represents approximately 40% of the Canadian population and 35% of Canada’s COVID-19 case load, such that the province’s epidemiology strongly influences that of Canada as a whole. Lastly, we assumed that attribution of COVID-19 deaths in Canada and comparator peer countries occurred in a comparable manner. The best available data (based on ratios of reported COVID-19 mortality to all-cause excess mortality during the pandemic) suggest that this is likely to have been the case for Canada, the US and France; reporting of COVID-19 mortality may have been more accurate in the UK than in Canada, which would tend to exaggerate the differences in outcomes between these two countries. More accurate reporting of COVID-19 deaths in Australia would lead us to underestimate the degree to which this country outperformed comparator peer countries ((37)).

## Conclusion

Canada’s relatively strong pandemic response during the first two years of the SARS-CoV-2 pandemic resulted in large numbers of deaths, hospitalizations and ICU admissions averted relative to responses in the US and UK, and more modest gains relative to France. A disease control stance focussed on elimination rather than mitigation, as was pursued in Australia during the same time period, would have resulted in further health and economic benefits.

## Appendix

**Table A1:** Mean and standard deviation for Oxford Pandemic Stringency Index in Canada and comparator peer countries, March 1, 2020 to May 1, 2022

Country	Mean	SD	*p*-value^a^
Canada	58.60	21.71	N/A
Australia	54.88	18.76	<0.001
France	48.84	21.08	<0.001
United Kingdom	51.14	24.05	<0.001
United States	53.12	17.98	<0.001

Abbreviations: N/A, not applicable; SD, standard deviation

^a^ *p*-value for Wilcoxon rank-sum test for comparison with Canada
